# Surgeons' beliefs and perceptions about removal of orthopaedic implants

**DOI:** 10.1186/1471-2474-9-73

**Published:** 2008-05-24

**Authors:** Beate Hanson, Chris van der Werken, Dirk Stengel

**Affiliations:** 1 AO Foundation, Clinical Investigation and Documentation, Dübendorf, Switzerland; 2 Division of Surgical Specialties, University Medical Centre Utrecht, Utrecht, The Netherlands; 3 Center for Clinical Research, Depts of Orthopaedic and Trauma Surgery, Unfallkrankenhaus Berlin and University of Greifswald, Germany

## Abstract

**Background:**

The routine removal of orthopaedic fixation devices after fracture healing remains an issue of debate. There are no evidence-based guidelines on this matter, and little is known on surgeons' practice and perceived effectiveness of implant removal in different clinical settings.

**Methods:**

A 41-item questionnaire was distributed to 730 attendees of the AO Principles and Masters Courses of Operative Fracture Treatment in Davos, Switzerland, to assess their attitudes towards removal of different types of implants, and perceived benefits and risks with this common procedure.

**Results:**

The response rate was 655/730 (89.7%), representing 54.6% of all 1199 course attendees. Surgeons from 65 countries (571 males and 84 females, mean age 39 ± SD 9 years) took part in the survey. Fifty-eight percent of the participants did not agree that routine implant removal is necessary, and 49% and 58% did not agree that indwelling implants pose an excess risk for fractures or general adverse effects. Forty-eight percent felt that removal is riskier than leaving the implant in situ. Implant removal in symptomatic patients was rated to be moderately effective (mean rating on a 10-point-scale, 5.8, 95% confidence interval 5.7–6.0). Eighty-five percent of all participants agreed that implant removal poses a burden to hospital resources. Surgeons were undetermined whether implant removal is adequately reimbursed by payers of health care services (44% "I-don't-know"-answers).

**Conclusion:**

Many surgeons refuse a routine implant removal policy, and do not believe in clinically significant adverse effects of retained metal implants. Given the frequency of the procedure in orthopaedic departments worldwide, there is an urgent need for a large randomized trial to determine the efficacy and effectiveness of implant removal with regard to patient-centred outcomes.

## Background

Implant removal belongs to the most common elective orthopaedic procedures in the industrial countries. In a frequently cited Finnish study, implant removal contributed to almost 30% of all planned orthopaedic operations, and 15% of all operations of the department [1].

Controversy exists as to the need for routine implant removal. In children, it may be necessary to remove implants early to avoid disturbances to the growing skeleton, to prevent their bony immuring making later removal technically difficult or impossible, and to allow for planned reconstructive surgery after skeletal maturation (e.g., in case of hip dysplasia).

In adults, pain, soft tissue irritation, the resumption of strenuous activities or contact sports after fracture healing, and the patient's demand are typical indications for implant removal in clinical practice. Many surgeons will remember patients whose intractable, hardly explainable local symptoms and complaints resolved quickly after the procedure. However, implant removal requires a second surgical procedure in scarred tissue, and poses a risk for nerve damage and re-fractures [2-5].

Pain may even worsen after implant removal. In a series of 109 femoral nail removals, an increase in pain and discomfort was noted in 4/58 (7%) of all patients with, and 10/51 (20%) of all patients without pre-operative symptoms [6]. Similar observations were made in subjects who had undergone open reduction and internal fixation of ankle fractures [7].

Corrosion, systemic release of nickel, chromium, and cobalt, and its presumed toxic, allergic, and even carcinogenic potential have been linked to stainless steel implants. As yet, none of these adverse effects had convincingly been confirmed in the clinical setting [8]. Orthopedic fixation devices made from titanium alloy are considered less susceptible to degradation and safe to be retained in situ [9,10], but titanium and aluminum had been traced in serum and hair of 16 of 46 patients after spinal instrumentation as well [11].

Little is known on the attitudes of orthopedic surgeons towards implant removal [12,13]. We reasoned that a questionnaire survey may be an appropriate method to evaluate surgeons' perceptions about the need, effectiveness, and risks of this common procedure in different clinical settings.

## Methods

We developed a three-page questionnaire with 41 items to determine surgeons' opinions and concerns about implant removal. As a first step, DS undertook a literature review in Pubmed Medline, Embase, SciSearch, and Google to identify available instruments assessing surgeons' beliefs and assumptions about implant removal and other common orthopedic procedures. We considered the items queried by Loder et al. in a recent web survey pragmatic and relevant for this study, and used them as a core set [12]. We then followed a sample-to-redundancy strategy to compile a comprehensive set of items describing the baseline profile of participants, potential confounders, and relevant endpoints [14-16]. The preliminary version of the questionnaire was emailed between the authors, who added, deleted, and revised individual items and questions until consensus was reached. The final version of the instrument was reviewed by three clinical experts (see acknowledgments), who felt comfortable with its overall extent, scope of questions, and scaling of possible answers.

The form contained three parts: 1) demographic information (including age, gender, level of training and specialization, current affiliation, and origin), 2) general beliefs about potential benefits and harms of retained material and removal surgery, and 3) reasons for removing implants (e.g., type of implant, clinical conditions demanding removal).

We requested participants to describe their practice of informing patients about the need for later implant removal at the time of fracture repair, and to estimate the influence of removal surgery on patients' complaints. Because cold welding may cause problems when attempting to remove interlocking plates, surgeons were asked how often they observe screw breakages, irremovable implant, and fractures with this frequently used material.

General beliefs were polled by 5-point Likert-scales. Answer options included "I strongly agree," "I strongly disagree," "I don't know," "I disagree," and "I strongly disagree." For all other questions, ratings were made on 10-point scales ranging from "1 = never" to "10 = always."

The survey was conducted during the last three days of the first, and the first three days of the second course week. A booth was established at the main entrance hall of the conference venue to allow participants for completing the questionnaire in a quiet and comfortable surrounding. Assistants addressed course attendants personally and invited them to participate in the survey. Also, posters were put up at main meeting places and the industrial exhibition explaining the goal of the study. As an incentive, all respondents took part in a drawing for an iPod^® ^player.

A consent waiver was granted by the Cantonal Ethics Board of Zurich. The participants were informed that, by filling out the questionnaire, they agreed in using the anonymously gathered data for research and publication.

All analyses were made in an exploratory intent, and we did not pose a formal null-hypothesis. The target sample size was planned to yield a certain precision of estimates, not to detect a relevant difference between groups with predefined type I and II errors [17]. A sample size of 650 eligible forms guaranteed standard errors ≤5% for proportions (in case of Likert-scale type answers) and continuous endpoints (i.e., 10-point scales). To compensate for a drop-out rate of 10%, a total of 730 forms were distributed during the meeting.

According to the quality of data, results are presented as proportions, means, or medians with their adequate measures of distribution and 95% confidence intervals (CI). For subgroup analyses (i.e., age, gender, profession, affiliation, origin), Likert-scale type ratings were analyzed by ordered logistic regression. Ten-point scales were analyzed by general linear models. To ease reading and data interpretation, the results from queries on general opinions about implant removal are expressed as proportions of disagreement (including "I strongly disagree" and "I disagree") and agreement (including "I strongly agree" and "I agree").

## Results

Of 730 distributed questionnaires, 655 (89.7%) were completed. This represented 54.6% of all 1199 attendees of the 2006 AO Principles and Masters Courses. Surgeons from 65 countries with a mean age of 38.8 ± SD 9.3 years (range, 26 to 71 years) took part in the survey. There were 571 males and 84 women. The demographic profile is summarized in Table 1.

**Table 1:** Demographic profile of the respondents.

**Characteristic**	
Mean age, years (SD)	38.8 (9.3)
Median age, years (range)	36 (26 – 71)
Gender	
Male	571 (87.2%)
Female	84 (12.8%)
Professional background	
General surgeon	96 (14.7%)
Orthopedic surgeon	361 (55.2%)
Trainee	121 (18.5%)
Spine surgeon	7 (1.1%)
Other	69 (10.6%)
Affiliation	
University hospital	277 (42.3%)
Public, non-university hospital	303 (46.3%)
Private hospital	61 (9.3%)
Other	14 (2.1%)
Current position	
Chief of staff	128 (20.0%)
Consultant	174 (27.1%)
Intern/resident	215 (33.5%)
Other	124 (19.3%)
Origin	
Africa	29 (4.5%)
Asia	58 (9.0%)
Australia	8 (1.2%)
Europe	470 (72.9%)
North America	38 (5.9%)
South America	42 (6.5%)

Table 2 summarizes the distribution of answers to questions about general opinions and attitudes. Many surgeons did not agree that orthopedic implants need to be routinely removed in younger, asymptomatic patients (disagreement: 57.8%, agreement: 37.1%). Also, many participants did not believe that indwelling implant pose an excess risk for fractures (disagreement: 48.7%, agreement: 35.2%) and allergy or malignancy (disagreement: 58.1%, agreement: 18.8%). Titanium was considered safer to be retained than stainless steel material (disagreement: 25.8%, agreement: 46.4%).

**Table 2:** Proportions of agreement and disagreement in statements about general attitudes towards implant removal.

**Statement**	**n**	**I strongly agree**	**I agree**	**I don't know**	**I disagree**	**I strongly disagree**
Orthopedic implants must be removed in younger patients (<40 years of age), even if they cause no problems	645	39 (6.0%) [4.3–8.2%]	200 (31.0%) [27.5–34.7%]	33 (5.1%) [3.5–7.1%]	297 (46.0%) [42.1–50.0%]	76 (11.8%) [9.4–14.5%]
Leaving implants in-situ poses a risk for later fractures	645	15 (2.3%) [1.3–3.8%]	212 (32.9%) [29.3–36.6%]	104 (16.1%) [13.4–19.2%]	287 (44.5%) [40.6–48.4%]	27 (4.2%) [2.8–6.0%]
Titanium implants are safer to be kept in-situ than devices made from stainless steel	647	48 (7.4%) [5.5–9.7%]	252 (38.9%) [35.2–42.8%]	180 (27.8%) [24.4–31.4%]	148 (22.9%) [19.7–26.3%]	19 (2.9%) [1.8–4.5%]
In case of otherwise unexplained pain and functional deficits, implant removal is a good option to improve the physical status	646	51 (7.9%) [5.9–10.2%]	394 (61.0%) [57.1–64.8%]	78 (12.1%) [9.7–14.8%]	111 (17.2%) [14.4–20.3%]	12 (1.9%) [1.0–3.2%]
Leaving implants in-situ increases the life-time risk for chronic infections, allergy, and cancer	649	15 (2.3%) [1.3–3.8%]	107 (16.5%) [13.7–19.6%]	150 (23.1%) [19.9–26.6%]	310 (47.8%) [43.9–51.7%]	67 (10.3%) [8.1–12.9%]
Removing implants is a surgical procedure which drains valuable hospital resources	644	114 (17.7%) [14.8–20.9%]	436 (67.7%) [63.9–71.3%]	21 (3.3%) [2.0–4.9%]	71 (11.0%) [8.7–13.7%]	2 (0.3%) [0.0–1.1%]
Removing implants damages healed soft tissue, and is riskier than retaining the device	643	29 (4.5%) [3.0–6.4%]	281 (43.7%) [39.8–47.6%]	75 (11.7%) [9.3–14.4%]	243 (37.8%) [34.0–41.7%]	15 (2.3%) [1.3–3.8%]
Given the time and efforts required for implant removal, this procedure is not adequately reimbursed by insurance companies	642	46 (7.2%) [5.3–9.4%]	190 (29.6%) [26.1–33.3%]	285 (44.4%) [40.5–48.3%]	112 (17.4%) [14.6–20.6%]	9 (1.4%) [0.6–2.6%]
Patients should take responsibility for the incurring costs of implant removal	642	15 (2.3%) [1.3–3.8%]	109 (17.0%) [14.2–20.1%]	91 (14.2%) [11.6–17.1%]	328 (51.1%) [47.1–55.0%]	99 (15.4%) [12.7–18.5%]

n = number of available answers. Percentages in brackets are 95% confidence intervals.

In contrast to the overall tendency against routine metal removal, 68.9% of all respondents agreed that it represents a therapeutic option in case of otherwise unexplained pain and functional deficits (disagreement: 19.0%). Interestingly, implant removal was considered only moderately effective in resolving local symptoms (mean rating 5.8, 95% CI 5.7 – 6.0).

Orthopedic surgeons were less enthusiastic about the appropriateness of implant removal in case of "symptomatic" implant than general surgeons. Both had similar opinions about the moderate effectiveness of this procedure.

A similar proportion of participants disagreed and agreed that implant removal causes additional soft tissue damage (40.1% versus 48.2%).

Most respondents considered implant removal a procedure that drains valuable hospital resources (agreement: 85.4%, disagreement: 11.3%).

Nearly half of all surgeons (44.4%) could not decide whether implant removal is adequately reimbursed by health care insurance carriers, and 36.8% and 18.8% agreed and disagreed that payments are inadequate for the procedure. However, most surgeons won't charge patients to pay for implant removal by themselves (disagreement: 66.5%, agreement: 19.3%).

**Figure 1:**
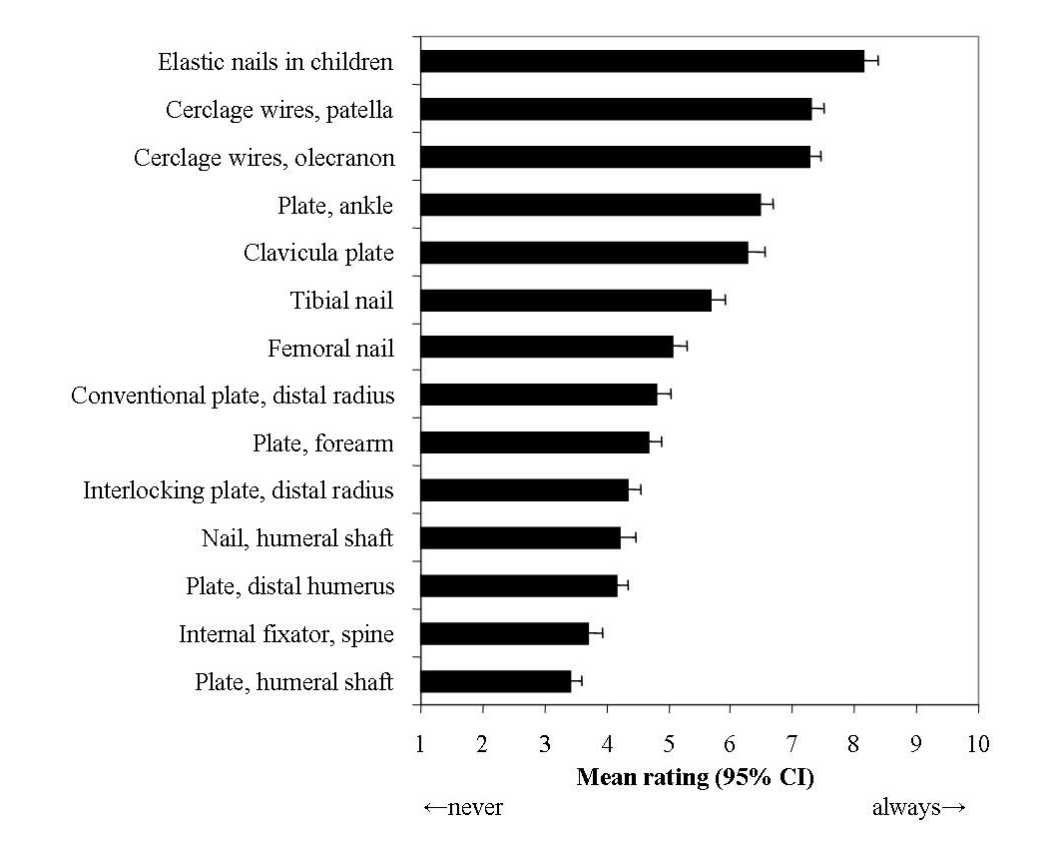
**Assigned removal priority to different types of implants.** Mean ratings on 10-point scales with 95% confidence intervals.

With a mean rating of 8.2 (95% CI 7.9 – 8.4) on a 10-point-scale, surgeons would recommend the regular removal of elastic titanium nails in children, followed by almost identical ratings for cerclage wires after fixation of fractures of the patella and the elbow (mean rating 7.3, 95% CI 7.1 – 7.5, mean difference 1.5 point, 95% CI 1.3 – 1.7 points, p < 0.0001). Plates at the humeral shaft were assigned the lowest priority for removal (mean rating 3.4, 95% CI 3.2 – 3.6). Findings are illustrated in Figure 1.

**Figure 2:**
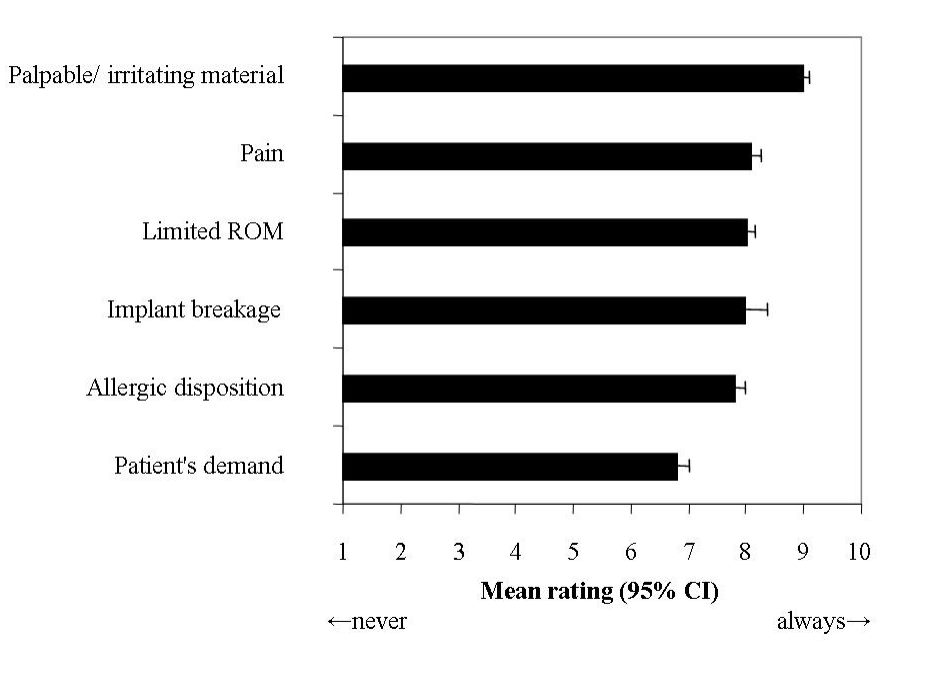
**Main indications for implant removal.** Mean ratings on 10-point scales with 95% confidence intervals.

Palpable and irritating material was considered the main indication for metal removal (mean rating 8.9, 95% CI 8.8 – 9.1), whereas the patient's demand ranked lowest on the list of potential indications (mean rating 6.8, 95% CI 6.6 – 7.0). Results are depicted in Figure 2.

Surgeons tended towards informing patients about the need for later implant removal at the time of fracture repair (mean rating 7.6, 95% CI 7.4 – 7.8).

Participants reported low rates of intra- and post-operative screw breakages (4.0, 95% CI 3.8 – 4.2), irremovable implants (3.0, 95% CI 2.9 – 3.2), and re-fractures (2.5, 95% CI 2.3 – 2.6).

Table 3 summarizes the findings from ordered logistic regression analysis. Older respondents, Europeans, and university-affiliated surgeons were more likely to agree that implants must be removed in younger patients. Titanium was considered safer to be retained than stainless steel by older participants, non-orthopedic surgeons, and Europeans. Also, non-orthopedic and European surgeons were more likely to agree that implant removal is a good therapeutic option to improve pain and function.

**Table 3:** Variables contributing to the likelihood of agreement in the different statements about general attitudes towards implant removal.

**Statement**	**Surgeons' age**	**Male *versus *female surgeon**	**Orthopedic *versus *non-orthopedic surgeon**	**European *versus *US-American origin**	**European *versus *non-European origin**	**University *versus *other affiliation**
Orthopedic implants must be removed in younger patients (<40 years of age), even if they cause no problems	1.03 (1.01–1.04)	1.12 (0.74–1.70)	1.23 (0.92–1.64)	7.40 (3.77–14.50)	1.56 (1.12–2.18)	0.72 (0.54–0.97)
Leaving implants in-situ poses a risk for later fractures	0.99 (0.98–1.01)	1.32 (0.86–2.02)	1.13 (0.84–1.50)	2.81 (1.44–5.47)	0.98 (0.71–1.35)	0.79 (0.59–1.06)
Titanium implants are safer to be kept in-situ than devices made from stainless steel	1.02 (1.00–1.04)	0.82 (0.54–1.22)	0.72 (0.54–0.96)	2.16 (1.18–3.97)	1.05 (0.76–1.45)	1.04 (0.78–1.38)
In case of otherwise unexplained pain and functional deficits, implant removal is a good option to improve the physical status	1.00 (0.98–1.02)	0.86 (0.53–1.38)	0.73 (0.54–1.00)	2.74 (1.47–5.11)	1.81 (1.28–2.55)	0.83 (0.61–1.13)
Leaving implants in-situ increases the life-time risk for chronic infections, allergy, and cancer	1.01 (0.99–1.03)	1.14 (0.74–1.76)	1.26 (0.94–1.68)	1.66 (0.90–3.08)	0.99 (0.72–1.37)	0.92 (0.69–1.22)
Removing implants is a surgical procedure which drains valuable hospital resources	1.00 (0.98–1.02)	1.51 (0.91–2.48)	0.99 (0.72–1.38)	1.39 (0.68–2.84)	1.11 (0.77–1.60)	1.08 (0.78–1.50)
Removing implants damages healed soft tissue, and is riskier than retaining the device	0.99 (0.97–1.00)	1.06 (0.69–1.64)	0.91 (0.68–1.22)	1.20 (0.65–2.23)	1.20 (0.65–2.23)	1.36 (1.01–1.82)
Given the time and efforts required for implant removal, this procedure is not adequately reimbursed by insurance companies	1.02 (1.00–1.03)	1.41 (0.93–2.14)	1.43 (1.07–1.91)	0.37 (0.19–0.70)	0.37 (0.19–0.70)	1.25 (0.93–1.67)
Patients should take responsibility for the incurring costs of implant removal	1.03 (1.01–1.05)	2.39 (1.52–3.76)	1.61 (1.20–2.16)	0.48 (0.26–0.91)	0.24 (0.17–0.34)	1.31 (0.98–1.75)

Numbers are odds ratios derived from ordered logistic regression, with 95% confidence intervals in parentheses. Values >1 indicate a higher likelihood of agreement, and values <1 indicate a higher likelihood in disagreement. If the 95% confidence limits exclude 1, statistically significant results can be assumed at the two-tailed 5% threshold.

Older, male, non-European, and university-based surgeons were more likely to agree that implant removal is inadequately reimbursed by health-care insurers.

## Discussion

In 1988, an estimated number of 4.9 million US American citizens had prevalent fixation devices [18]. With expanding indications for operative fracture treatment in all age groups and fracture types, it is likely that this number has substantially increased during the last two decades.

The findings from this survey indicate that about 60% of all surgeons do not agree in a routine removal policy in asymptomatic subjects, and that the patient's request is the less important reason to remove material. Many surgeons doubt clinically significant adverse effects of indwelling metal like stress shielding or an allergic or even carcinogenic potential [19-22], and acknowledge the burden of implant removal to hospital resources.

While there are absolute (e.g., cartilage damage by intra-articular screws, skin and soft tissue irritation by prominent material) and relative indications to take out implant (e.g., to reduce artifacts with planned MRI and CT imaging), the discrepancy between physical findings, imaging results and complaints can be remarkable. Given the apparently quick and safe removal procedure, many surgeons may be tempted to take their patients to the operating theatre instead of conservative management or watchful waiting.

There is currently no controlled trial that would allow for a valid trade-off between the benefits and harms of implant removal, and scientifically grounded counseling of patients. In addition to the possibility of retained material and another period of sick leave and restricted weight bearing, patients must be informed about potential risks of the removal operation [6,8,23-25]. In a review of 14 studies enrolling 635 patients who underwent removal of forearm plates, the overall incidence of complications ranged from 12 to 40% [26]. Iatrogenic nerve injuries were noted in 2 to 29%, refractures in 2 to 26%, and wound infections in 5 to 12% of all studies.

According to eight retrospective studies enrolling 346 symptomatic patients (Table 4), the weighted success rate (i.e., a complete or marked reduction of pain) of implant removal can be estimated at 78%. On the other hand, the weighted failure rate (including subjects with worsening pain) is 22%, or about 1 in 5 patients. In a recent prospective study of 60 patients, mean pain scale, Short Musculoskeletal Function Assessment (SMFA), and Short Form 36 Physical Component Scores (SF36-PCS) significantly improved over one year after removal of painful implants [25]. With 13 different anatomic sites, and a mean in-situ time ranging from six months to nine years, unspecific effects of removal surgery may have contributed to the observed outcomes. The lack of a head-to-head comparison in all available studies prohibits conclusive inferences about a causal relationship between implant removal and symptom control.

**Table 4:** Uncontrolled studies of the effectiveness of implant removal in symptomatic patients. Success includes complete or marked reduction of pain

**Author**	**Year**	**Implant**	**n**	**Success**		**Failure**	
		
Richards [30]	1992	various	46	42	91% (79 – 98%)	4	9% (2 – 21%)
Jacobsen [31]	1994	ankle plates	66	49	74% (62 – 84%)	17	26% (16 – 38%)
Court-Brown [32]	1997	intramedullary tibial nails	62	60	97% (88 – 99%)	2	3% (0 – 11%)
Dodenhoff [33]	1997	intramedullary femoral nails	17	11	65% (38 – 86%)	6	35% (14 – 62%)
Keating [34]	1997	intramedullary tibial nails	49	39	80% (66 – 90%)	10	20% (10 – 34%)
Brown [7]	2001	ankle plates	22	11	50% (28 – 72%)	11	50% (28 – 72%)
Gösling [6]	2004	intramedullary femoral nails	58	45	78% (65 – 87%)	13	22% (13 – 35%)
Gösling [24]	2005	intramedullary tibial nails	26	19	73% (52 – 88%)	7	27% (12 – 48%)
							
Weighted average			346		78% (69 – 88%)		22% (12 – 31%)

Failure includes no change and worsening of symptoms. Summary estimates were derived from random-effects meta-regression analysis

Elastic stable intramedullary nails (ESIN) ranked first among all implants to be considered for routine removal. This information may add to results from a recent survey of 273 pediatric and 99 non-pediatric specialists regarding implant removal in children [12]. While 64% and 50% of all respondents recommended removing DCPs after upper extremity fractures and interlocking nails after femoral shaft fractures, the different scenarios did not offer an ESIN option. Some authors admitted their inclination to remove flexible nails in children despite the lack of scientific evidence and the potential risk for refractures [27,28].

Several limits of this investigation merit discussion. First, as a survey, it can only describe opinions and practice patterns, and does not allow for determining the actual effectiveness of implant removal. Questionnaire surveys are prone to multiple sources of bias (e.g., socially desired response bias) [29], and answers of the respondents may not reflect their true daily behaviour. In addition, recalled numbers may be incorrect. Recall bias may also have been introduced by remembering a recent successful or unsuccessful case. Second, European professionals represented the largest fraction of all respondents, thereby restricting the external validity of the findings. Third, the survey form may have missed certain scenarios, and some of the questions may also be regarded ambiguous or sketchy.

We tried hard to cover a broad range of clinical settings, and to enrol a large and possibly representative sample of surgeons. Also, the response rate and the number of completed items make the estimates reliable. Apart from all design limitations, the findings may point towards a true public health problem. Since most surgeons are reluctant in charging patients for the procedure, reimbursement strategies need to be evaluated and adopted to the substantial time and effort associated with implant removal surgery.

## Conclusion

There is a serious need to study the biological mechanisms and clinical determinants of symptomatic implants, and to develop clinical decision rules that may allow for identifying patients who will benefit most from implant removal. A controlled trial that compares removal to retention is strongly warranted.

## Competing interests

The authors declare that they have no competing interests.

## Authors' contributions

BH and DS had the idea for this study, developed the design, and designed the questionnaires. CvdW made substantial comments on both the study design and the questionnaires justifying authorship. DS performed all statistical analyses. BH and DS jointly drafted the manuscript. All authors read and approved the final version of this paper.

## Pre-publication history

The pre-publication history for this paper can be accessed here:


